# Municipal Appointments Health Department

**Published:** 1881-01

**Authors:** 


### MUNICIPAL APPOINTMENTS, HEALTH DEPARTMENT.

The time is near at hand for the annual appointment of Health and District Health Physicians. These positions are both important and responsible, and a judicious discrimination should be exercised in filling them. As medical journalists we feel it our duty to direct attention to this subject, and to join in the hope that professional fitness rather than political influence will determine the choice. During the past year the city has been fortunate in its Health Physician, Dr. A. H. Briggs, who has proven an energetic and capable sanitary officer, securing the confidence of the profession, and also of the public. The Board of Health, if it consults the interests of the city, will retain the services of this official, whose heart and hand have been so actively and earnestly engaged during the year in behalf of the public health.

We can but express the wish that judicious selections will be made for the subordinate positions. Many of our rising young men seek these places not only for the pecuniary compensation offered, but for the experience acquired in the professional care of the poor, for whose special benefit they are appointed. In this matter our local philanthropists are deeply interested. The deserving poor, if prostrated with disease, need skillful treatment as well as kind and considerate care, objects designed to be attained through our charitable societies. The city aims to provide professional attendance and medicine, as well as food and sustenance, if necessity demands. It should be as discriminating in the selection of the former, as in the quality of the latter. We are well assured that the enlightened sentiment of the community will warmly endorse the appointment of the best talent among the junior practitioners for the important positions of District Health Physicians.

